# The neuronal and astrocytic protein SLC38A10 transports glutamine, glutamate, and aspartate, suggesting a role in neurotransmission

**DOI:** 10.1002/2211-5463.12219

**Published:** 2017-04-26

**Authors:** Sofie V. Hellsten, Maria G. Hägglund, Mikaela M. Eriksson, Robert Fredriksson

**Affiliations:** ^1^ Department of Pharmaceutical Bioscience, Molecular NeuropharmacologyUppsala UniversitySweden; ^2^ Department of Neuroscience, Functional PharmacologyUppsala UniversitySweden

**Keywords:** glutamate/GABA‐glutamine cycle, glutamine transport, immunohistochemistry, SLC38A10, solute carriers

## Abstract

In brain cells, glutamine transporters are vital to monitor and control the levels of glutamate and GABA. There are 11 members of the SLC38 family of amino acid transporters of which eight have been functionally characterized. Here, we report the first histological and functional characterization of the previously orphan member, SLC38A10. We used pairwise global sequence alignments to determine the sequence identity between the SLC38 family members. SLC38A10 was found to share 20–25% transmembrane sequence identity with several family members, and was predicted to have 11 transmembrane helices. SLC38A10 immunostaining was abundant in mouse brain using a custom‐made anti‐SLC38A10 antibody and colocalization of SLC38A10 immunoreactivity with markers for neurons and astrocytes was detected. Using *Xenopus laevis* oocytes overexpressing SLC38A10, we show that SLC38A10 mediates bidirectional transport of l‐glutamine, l‐alanine, l‐glutamate, and d‐aspartate, and efflux of l‐serine. This profile mostly resembles system A members of the SLC38 family. In conclusion, the bidirectional transport of glutamine, glutamate, and aspartate by SLC38A10, and the immunostaining detected in neurons and astrocytes, suggest that SLC38A10 plays a role in pathways involved in neurotransmission.

**Abbreviations**
EAAT: excitatory amino acid transporter
GGGC: glutamate/GABA‐glutamine cycle
IHC: immunohistochemistry
mTORC1: mechanistic target of rapamycin complex 1
SLC: solute carrier
SNAT: sodium‐coupled neutral amino acid transporter
TMHs: transmembrane helices

Membrane‐bound transporters are responsible for uptake and efflux of solutes, for example, amino acids, in cells and organelles 1. In the central nervous system, amino acid transporters also play an important role as transporters for neurotransmitters, for example, glutamate and aspartate and precursors for neurotransmitter synthesis 2. Furthermore, amino acid transporters can also function as nutrient sensors, and because of their transport and sensing functions they can control the protein synthesis, by either increase or decrease synthesis, depending on nutrient availability 3, 4.

The Solute carrier (SLC) superfamily is the largest group of transporters in the human genome, and up to date, 395 SLCs are identified and divided into 52 families, with different biochemical properties 1. The members in each SLC family have at least 20% amino acid sequence identity to another member in the family 5. SLC transporters are membrane‐bound, located to the plasma membrane 6, vesicles 7, lysosomes 8, peroxisomes 9, and mitochondria 10, and function as ATP‐independent uniporters, symporters, or antiporters. SLCs have a wide substrate profile and mediate flux of for example amino acids, ions, drugs, nucleotides, fatty acids, and vitamins 1.

The SLC38 family, the system A and system N sodium‐coupled neutral amino acid transporter family, has 11 members, encoded by the genes, *SLC38A1–SLC38A11* 11. The characterized members are termed SNAT# (SLC38A#), Sodium coupled Neutral Amino acid Transporter# 12, and up to date, eight members are functionally characterized. The transporters in the family are divided into system A or system N, depending on transport characteristics 11. System A transporters have a wider substrate profile and transport small neutral amino acids, in particular glutamine, alanine, and serine 13. The system N transporters have a more restricted substrate profile, and translocate mainly glutamine, asparagine, and histidine 14. Both the system A transporter SLC38A2 and the system N transporter SLC38A5 are pH dependent, with highest activity at pH 8 15. However, the transport mechanism differs. System A transport of amino acids is sodium coupled 16, while the movement of amino acids by system N is sodium coupled with hydrogen in exchange 17. Furthermore, only system A transporters are affected by the amino acid analog Metyl‐amino‐isobutyric acid (MeAIB) 18. Glutamine is a preferred substrate throughout the family 11, except for SLC38A4, which mainly transports alanine, asparagine, and cysteine 19. SLC38A1 20, SLC38A2 21, SLC38A4 19, and SLC38A8 22 are characterized to system A, while SLC38A3 17, SLC38A5 23, and SLC38A7 24 are classified to system N. SLC38A9 is not classified into any system yet, but was recently characterized as a component of the amino acid‐sensing Ragulator–Rag complex on lysosomes, controlling the activation of the mechanistic target of Rapamycin complex 1 (mTORC1) in response to amino acids 8, 25, 26 and shown to transport glutamine, arginine, and asparagine 8.

In the brain, SLC38A1 20, 27, 28, 29 and SLC38A2 30 are expressed in both neurons and astrocytes, while SLC38A3 31 and SLC38A5 32 are selectively astrocytic, and SLC38A6 33 SLC38A7 24, SLC38A8 22, and SLC38A9 34 are selectively neuronal. Several SLC38 transporters are suggested to participate in the glutamate/GABA‐glutamine cycle (GGGC), which takes place between neurons and astrocytes 11, 35. Glutamate is a potent neurotoxin, and it is vital to have a system for glutamate reuptake, following release by excitatory neurons in the synaptic cleft. Glutamate is produced from glutamine hydrolysis catalyzed by glutaminase in excitatory neurons, and further used as a precursor for GABA in inhibitory neurons 36. Glutamate released by excitatory neurons is taken up by astrocytes and converted to glutamine, by the enzyme glutamine synthetase. Glutamine is then released from astrocytes and taken up by neurons, completing the cycle 37. SLC38A1 16, 38, SLC38A2 38, SLC38A7 24, and SLC38A8 22 are suggested to facilitate the uptake of glutamine in neurons, and SLC38A3 39 and SLC38A5 40 the efflux of glutamine from astrocytes.

In rat, *Slc38a10* mRNA expression was detected throughout the body, with highest relative expression in pituitary, eye, and lung 41. In another study, where the gene expression of SLCs in rat gastrointestinal (GI) tract was studied, *Slc38a10* and *Slc38a2* were the only SLC38 family members expressed. In addition, they were ubiquitously expressed along the GI tract, suggesting a potential role for SLC38A10 in absorption of amino acids from the GI tract 42.

Here, we report bioinformatics data, as well as histological and functional data, of the previously orphan member SLC38A10. We have used pairwise global sequence alignments to determine the sequence identity between the SLC38 family members and predicted the transmembrane helices of SLC38A10. The immunostaining of SLC38A10 was mapped in the mouse brain using nonfluorescent immunohistochemistry and fluorescent immunohistochemistry was used to determine colocalization of SLC38A10 staining with specific cell markers. In addition, *Xenopus laevis* oocytes were used to overexpress the transporter in order to determine substrate profile for SLC38A10.

## Materials and methods

### Predictions of transmembrane helices and global sequence alignments

The full FASTA human protein sequences for all SLC38 family members were downloaded from the NCBI webpage, [SLC38A1 (NP_001265318.1), SLC38A2 (NP_061849.2), SLC38A3 (NP_006832.1), SLC38A4 (NP_001137296.1), SLC38A5 (NP_277053.2), SLC38A6 (NP_001166173.1), SLC38A7 (NP_060701.1), SLC38A8 (NP_001073911.1), SLC38A9 (NP_775785.2), SLC38A10 (NP_001033073.1), and SLC38A11 (NP_001186077.1)]. The sequence identity was analyzed using EMBOSS Needle global sequence alignment tool (http://www.ebi.ac.uk/Tools/psa/emboss_needle/). The full protein sequences were pairwise aligned between all members in the family. Furthermore, also the transmembrane helices (TMHs) sequences were pairwise aligned between SLC38A10 and the other family members. The predictions of the TMHs were performed using the tmhmm server v.2.0 (http://www.cbs.dtu.dk/services/TMHMM/).

### Ethical statement

All animal procedures for C57Bl6/J mice (Taconic M&B, Lille Skensved, Denmark) and *X. laevis* frogs (Nasco, Fort Atkinson, WI, USA) were approved by the local ethical committee in Uppsala following the guidelines of the European Communities Council Directive (2010/63), (permit number, mice; C188/12 and frogs; C98/13). Adult male mice were used in all experiments including mice and the animals were kept in a temperature controlled room on a 12‐h light–dark cycle with unlimited access to food, R3 chow (Lantmännen, Stockholm, Sweden) and water. Oocyte‐positive female African claw frogs, *X. laevis*, were used in all transport experiments including oocytes and the frogs were housed in a temperature controlled room at 21 °C, on a 12‐h light–dark cycle with access to salmon pellets (Aller aqua, Christiansfeld, Denmark).

### Western blot

Protein supernatant was extracted from an adult, male C57Bl6/J mouse. In short, crude protein was extracted by homogenizing 0.2 mg brain tissue in 1 mL of lysis buffer [50 mm Tris‐HCl pH 8, 150 mm NaCl, 4 mm MgCl, 0.5 mm EDTA, 2% Triton X‐100, and 1 mm Protease inhibitor PMSF diluted in isopropanol (Sigma‐Aldrich, St. Louis, MO, USA)]. After a short centrifugation, the supernatant was transferred to a new tube. Subsequently, the homogenate was centrifuged at 10 000 ***g*** for 10 min receiving pellets and supernatant. Protein concentrations were determined by protein assay DC according to manufacturer's instructions (Bio‐Rad Laboratories, Hercules, CA, USA). Lammlis loading buffer (Bio‐Rad Laboratories) and DTT (in a 4 : 1 ratio) were added to protein supernatant samples (60 μg) prior denaturation by heating to 95 °C for 5 min. Samples and PageRuler prestained protein ladder (Fermentas, Burlington, ON, Canada) were separated on a Mini‐Protean TGX gel (4–10%, Bio‐Rad, Hercules, CA, USA) by gel electrophoresis run at 150V for 30 min in running buffer (0.1% SDS, 0.025 m Tris base and 0.192 m glycine). The gel was transferred to an Immobilon‐P polyvinylidene fluoride (PVDF) membrane (Millipore, Billerica, MA, USA) and run at 100V for 55 min in transfer buffer (0.025 m Tris base, 0.192 m glycine and 20% methanol). The membrane was preblocked for 1 h in blocking buffer [5% nonfat dry milk (Bio‐RAD) diluted in 1.5M NaCl, 0.1M Tris, 0.05% Tween‐20, pH 8.0 (TTBS)] and then incubated in primary antibody against SLC38A10 (diluted 1 : 800, rabbit‐anti‐SLC38A10, synthetic peptide sequence (NH_2_‐)MTAASTSKWGLIC(‐CONH_2_), Innovagen, Lund, Sweden) overnight at 4 °C. After washes in TTBS, the membrane was incubated in HRP‐conjugated secondary antibody (diluted 1 : 10 000, goat‐anti‐rabbit, Invitrogen, Waltham, MA, USA) for 1 h, washed again in TTBS followed by detection with the enhanced chemiluminescent (ECL) method and developed on High performance chemiluminescence film (GE healthcare, Chicago, IL, USA).

### Tissue collection and sectioning

Mice were anesthetized with an intraperitoneal injection of sodium pentobarbital (9 mg·kg^−1^ IP; Apoteksbolaget, Stockholm, Sweden). Transcardial perfusion was performed through the left ventricle with phosphate‐buffered saline (PBS) followed by 4% formaldehyde (HistoLab, Gothenburg, Sweden) fixation. Brain and spinal cord tissues were surgically removed and stored in 4% formaldehyde overnight. For free‐floating brain tissue sections, the tissue was washed in PBS, embedded in 4% agarose and sections were cut at 70 μm by using a vibratome (Leica VT1000S Microsystems, Wetzlar, Germany) in PBS. For paraffin embedding, tissues were fixed in zinc‐formalin (Thermo Fisher, Waltham, MA, USA), dehydrated, and paraffin infused (Tissue‐Tek vacuum infiltration processor, Sakura, the Netherlands). Paraffin sections of 7 μm were cut using a Microm 355S STS cool cut microtome (Thermo Fisher).

### Nonfluorescent immunohistochemistry on free‐floating sections

Single immunohistochemistry (IHC) was performed with the primary SLC38A10 antibody (diluted 1 : 200, rabbit‐anti‐SLC38A10, Innovagen) and a biotinylated secondary antibody (diluted 1 : 400, goat‐anti‐rabbit, Vector Laboratories, Burlingame, CA, USA) as previously described 43.

### Fluorescent immunohistochemistry on paraffin‐embedded sections

Fluorescent IHC was performed on paraffin‐embedded mouse brain sections according to Hägglund *et al*. 24, with the following changes. The primary SLC38A10 antibody was combined with antibody markers detecting NeuN, pan Neuronal, MAP2, GAD67, GFAP, pan Cytokeratin, and Synaptophysin overnight at 4 °C. For antibody information see Table 1. Fluorescent staining was analyzed using a fluorescent microscope (Zeiss Axioplan2 imaging) connected to a camera (AxioCam HRm) with the axiovison 4.7 software.

**Table 1:** Details of antibodies used for fluorescent immunohistochemistry

	Species	Dilution	Company
Primary antibodies			
Anti‐SLC38A10	Rabbit	1 : 400	Innovagen, Sweden
Anti‐NeuN	Mouse	1 : 400	Millipore, Solna, Sweden
Anti‐Pan Neuronal	Mouse	1 : 100	Millipore, Sweden
Anti‐MAP2	Mouse	1 : 500	Sigma, Malmö, Sweden
Anti‐Gad67	Mouse	1 : 200	Millipore, Sweden
Anti‐GFAP	Chicken	1 : 400	Abcam, Cambridge, UK
Anti‐Pan Cytokeratin	Mouse	1 : 200	Sigma, Sweden
Anti‐Synaptophysin	Mouse	1 : 200	BD Transduction Laboratories, Stockholm, Sweden
Secondary antibodies			
Anti‐rabbit‐594	Donkey	1 : 400	Invitrogen, USA
Anti‐mouse‐488	Goat	1 : 400	Invitrogen, USA
Anti‐chicken‐488	Goat	1 : 400	Invitrogen, USA

### Cloning of Slc38a10 and cRNA synthesis for oocyte microinjections

The mouse *Slc38a10* clone (6493546) was purchased from Invitrogen. *Slc38a10* was amplified using the forward (GATCGAATTCACAGCCACCGGAATGACGGCCGCCTCCACCTCC) and reverse (GATCGCGGCCGCTCAGGCTTCCTCCAGATCTCCAG) primers and Platinum proofreading DNA polymerase (Invitrogen) according to standard PCR procedures. *Slc38a10* was cloned using the TOPO^®^‐TA cloning kit (Invitrogen) following the manufacturers protocol. *Slc38a10* was again linearized and amplified using the forward reverse primers, previous listed. The oocyte vector pβGFP/RN3P and the *Slc38a10* PCR product were cut using the enzymes EcoRI and NotI (Fermentas) and ligated together using T4 ligase (Invitrogen). Heat chock transformation of the pβGFP/RN3P/*Slc38a10* construct was performed followed by colony PCR using T7 and T3 primers. Plasmid cDNA was prepared with the JET star 2.0 Plasmid Purification Midi kit/50 (Genomed, Germany) and the concentration was measured with Nanodrop^®^ ND‐1000 Spectrophotometer (Nanodrop technologies, Wilmington, DE, USA). The clone was sequenced (Eurofins MWG Operon, Ebersberg, Germany) and verified correct. The *Slc38a10* cRNA was synthesized by digesting the pβGFP/RN3P/*Slc38a10* vector with SfiI (Fermentas). The mMessage mMachine^®^ T3 transcription kit (Thermo Fisher) and the PCR purification kit (Qiagen, Venlo, the Netherlands) were used for capped RNA transcription and purification following the manufacturer's protocol. The cRNA concentration was measured and controlled using the Experion RNA Std‐Sens Analysis kit with an Experion automated electrophoresis system (Bio‐Rad). The cRNA was stored at −80 °C.

### Oocyte preparation and oocyte microinjections

*Xenopus laevis* frogs were anesthetized using a 2.5 g·L^−1^ MS222 [Ethyl 3‐aminobenzoate methanesulfonate salt, (Sigma‐Aldrich)] bath buffered with NaHPO_4_ (Sigma‐Aldrich) to pH 7 for 10–15 min. Oocytes were harvested from ventrum and treated with seven units of Liberase™ (Roche Diagnostics, Basel, Switzerland) for 2 h at 19 °C, rotating 90° 2 rpm. Stage V and VI oocytes were used for experiments and oocytes were injected with 23 nL *Slc38a10* cRNA (15–23 ng) using the Nanoject II autonanoliter injector (Drummond Scientific, USA). The injected and noninjected control oocytes were incubated in OR2 buffer (82.0 mm NaCl, 2.5 mm KCl, 1.0 mm CaCl_2_, 1.0 mm MgCl_2_, 1.0 mm Na_2_HPO_4_, and 5.0 mm HEPES, pH 7.8) supplemented with 67 μg·mL^−1^ tetracycline hydrochloride (MP Biomedicals, Santa Ana, CA, USA) for 2–4 days at 19 °C prior experiments.

### Radiolabeled uptake assays

Radiolabeled uptakes assays were performed as previously described in 24. Briefly, oocytes were incubated in KRH transport buffer (118.0 mm NaCl, 4.8 mm KCl, 2.5 mm CaCl_2_, 10.0 mm Hepes, pH 7.8) for 15 min prior to incubation in 100 μL of radiolabeled amino acid (0.2 μm labeled and 1.0 μm nonlabeled l‐amino acid in KRH, for d‐Asp; 0.2 μm [^3^H]‐d‐Asp and 1.0 μm nonlabeled l‐Asp was used) for 50 min or every 5 min for 60 min in 96‐well BRANDplates^®^ pureGrade™ (BRAND GMBH, Wertheim, Germany). Then 100 μL of ice cold KRH buffer was added to stop the uptake and the oocytes were washed twice in 200 μL of ice cold KRH buffer. Oocytes were lysed in 100 μL 10% SDS and transferred to scintillation tubes Minis 2000 (Zinsser analytic, Frankfurt, Germany) with 2.5 mL Aquasafe 300 plus scintillation liquid (Zinsser analytic). The uptake was measured in counts per minute (cpm) using the liquid scintillation analyzer, Tri‐Carb^®^ 2910 TR (Perkin Elmer, Waltham, MA, USA). Uptake experiments were performed with *n* = 5–12 oocytes in each group, (SLC38A10 overexpressing oocytes and control oocytes) and outliers were removed using Grubbs test. All amino acids used were labeled with [^3^H], and MeAIB was [^14^C] labeled, all purchased from Perkin Elmer with specific activity, l‐Ala 83.0 Ci·mmol^−1^, l‐Arg 43.0 Ci·mmol^−1^, d‐Asp 12.9 Ci·mmol^−1^, l‐Glu 51.1 Ci·mmol^−1^, l‐Gln 50.3 Ci·mmol^−1^, Gly 52.0 Ci·mmol^−1^, l‐Leu 56.2 Ci·mmol^−1^, l‐Lys 97.3 Ci·mmol^−1^, l‐Met 81.0 Ci·mmol^−1^, l‐Pro 75.0 Ci·mmol^−1^, l‐Ser 15.0 Ci·mmol^−1^, l‐Tyr 40.0 Ci·mmol^−1^, and MeAIB 58.7 mCi·mmol^−1^. All nonlabeled amino acids were purchased from Sigma‐Aldrich.

### Radiolabeled efflux assays

Fourteen oocytes (seven SLC38A10 and seven control oocytes) were injected with 23 nL [^3^H]‐labeled amino acid and incubated in ice cold ND96 transport buffer (96.0 mm NaCl, 2.0 mm KCl, 1.8 mm CaCl_2_, 1.0 mm MgCl_2_, 5.0 mm Hepes, pH 7.8 or pH7.4) for 15 min on ice followed by two washes in 2 mL ice cold ND96 buffer in 24‐well tissue culture plates (VWR, Radnor, PA, USA). The oocytes were incubated in 1 mL RT ND96 buffer and 100 μL of buffer was extracted from the supernatant in each well every 5 min for 30 min and transferred to scintillation tubes Minis 2000 (Zinsser analytic) with 2.5 mL Aquasafe 300 plus scintillation liquid (Zinsser analytic). The efflux was measured in cpm with the liquid scintillation analyzer, Tri‐Carb^®^ 2910 TR (Perkin Elmer). The oocyte and the remaining 400 μL ND96 buffer in each well after the time course efflux experiment was also analyzed using scintillation counting to facilitate discovering of outliers, either by failed amino acid injection or leaking oocytes. Outliers were removed with Grubbs test after evaluation of the measured cpm in the oocyte and the remaining buffer following the efflux assay. Each analyzed experiment presented has *n* = 5–7 oocytes in each treatment group.

### Statistical analysis

All graphs and statistical analysis were performed using the software graphpad prism 5. Unpaired t‐tests with 95% confidence interval was performed between oocytes overexpressing SLC38A10 and control oocytes, (*P < 0.05, **P < 0.01, ***P < 0.001).

## Results

### Prediction of transmembrane helices for SLC38A10

**Figure 1:**
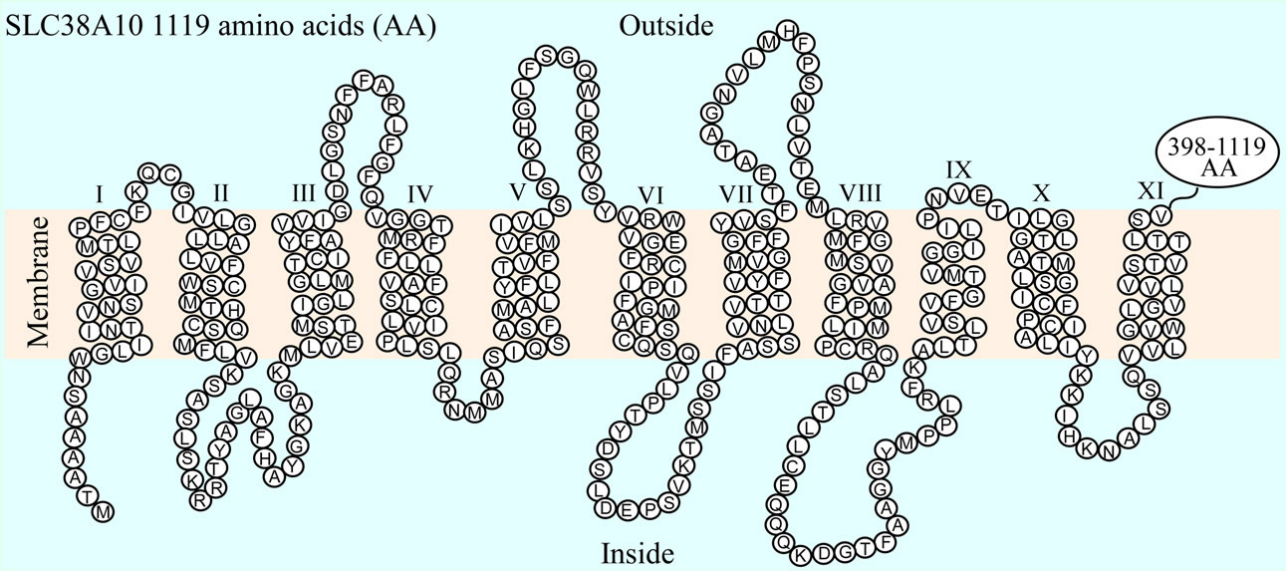
Predicted transmembrane folding of SLC38A10. The transmembrane helices were predicted for the human SLC38A10 protein sequences. Eleven TMHs were identified (I‐XI). The circles represent each amino acid (AA) in the protein sequence and the last 398–1119 amino acids are not specified. SLC38A10 was found to have an intracellular N‐terminal and a long C‐terminal of 722 amino acids outside the membrane.

The human SLC38A10 protein sequence was analyzed to predict the transmembrane helices (TMHs) of the protein. According to the TMHMM Server v.2.0, nine helices (I–V, VII, and IX–XI), that fulfill the criteria's to be TMHs were found, and additional two (VI and VIII) TMHs was manually identified by the highest score of membrane localization. This analysis revealed that SLC38A10 has an intracellular N‐terminal and a long C‐terminal of 722 amino acids (amino acid 398–1019) on the outside of the membrane. A model of the predicted transmembrane folding of the SLC38A10 protein sequence with 11 TMHs is presented in Fig. 1.

### Matrix of global sequence alignments for the SLC38 family

**Figure 2:**
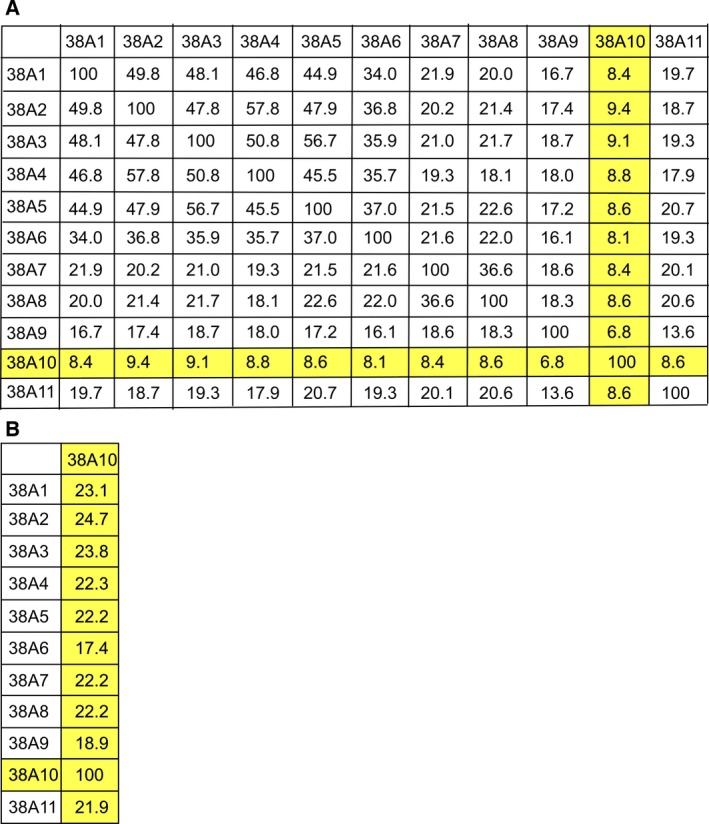
Sequence identity matrixes of the SLC38 family members. EMBOSS global sequence alignment tool was used to align the human protein sequences for the SLC38 family members. (A) The 12 × 12 matrix display the sequence identity in % between all members in the family. The full human protein sequence for all members was used. SLC38A10 had low sequence identity, <10%, with the other members, because of the length of the protein sequence, which is approximately twice as long as for the other members. (B) The amino acids from the first to the last transmembrane helices were identified for all members, and pairwise aligned with the transmembrane sequence for SLC38A10. This matrix shows that SLC38A10 share high transmembrane sequence identity with most of the members in the SLC38 family. SLC38A10 shares more than 20% sequence identity with all members in the family, except with SLC38A6 and SLC38A9.

The full human protein sequences for the SLC38 family members were pairwise aligned using EMBOSS Needle global sequence alignment tool. The complete protein sequences were pairwise aligned between all SLC38 family members and the sequence identity between the members is presented in percent (%) (Fig. 2A). Overall, this analysis displays that SLC38A1‐A6 have high sequence identity ranging from 34–58%, while only some of these members share approximately 20% sequence identity with SLC38A7, SLC38A8, and SLC38A11. However, SLC38A7 and SLC38A8 share 37% sequence identity. Moreover, this analysis displays that both SLC38A9 and SLC38A10 have less than 20% sequence identity with the other family members. For SLC38A10, the sequence identity is less than 10% with all other family members, and hence, it does not fulfill the criteria (>20%) for being a SLC38 family member. The protein sequence of SLC38A10 is more than twice as long as the other members of the SLC38 family, with a long C‐terminal tail without homology to any other member of the family. Therefore, the global sequence alignment was performed again, with only the protein sequence from start of TMH 1 until end of TMH 11 was used (Fig. 2B). This analysis showed that SLC38A10 share more than 20% sequence identity with all members except for SLC38A6 and SLC38A9, with highest sequence identity to SLC38A2 (24.7%). In general, this analysis displays that SLC38A10 share sequence identity within the range of 21.9–24.7% with all the other family members.

### Western blot to verify the custom‐made anti‐SLC38A10 antibody

**Figure 3:**
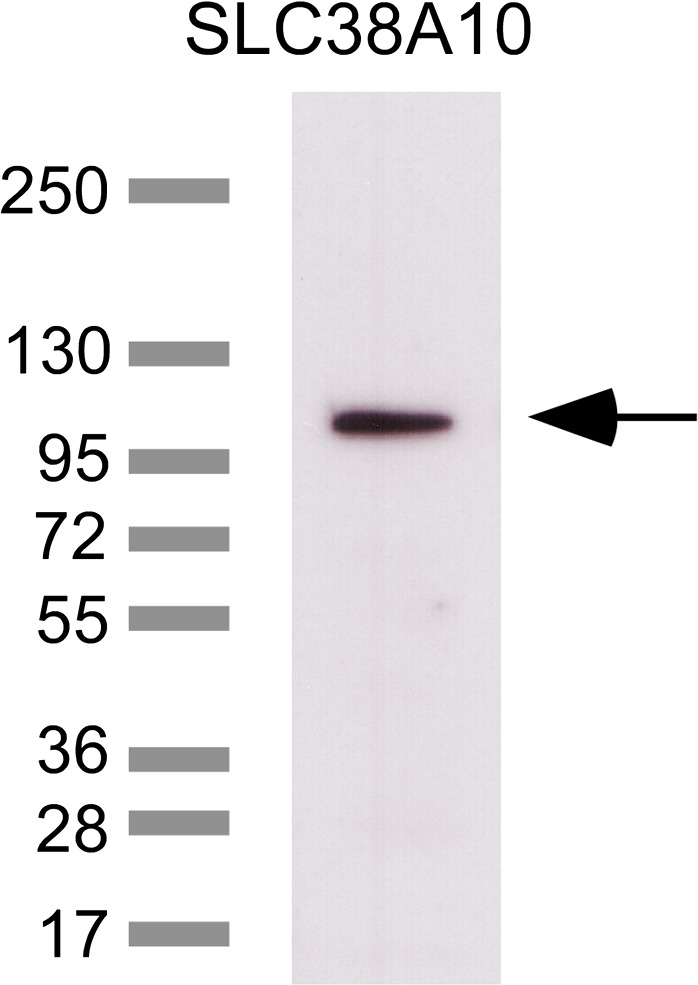
Verification of the anti‐SLC38A10 antibody specificity. Characterization of the custom‐made polyclonal SLC38A10 antibody was performed with western blot. The western blot shows a supernatant (60 μg) crude lysate fraction and the ladder in kDa. One strong band was detected at ~110 kDa, suggesting specificity of the custom‐made polyclonal SLC38A10 antibody.

We generated a rabbit polyclonal anti‐SLC38A10 antibody. The specificity of the custom‐made antibody was verified using western blot analysis (Fig. 3). The theoretical size of the SLC38A10 protein in mouse is 116.3 kDa (AAH82300.1, 1081 amino acids). The western blot detected a strong band at approximately 110 kDa, suggesting epitope‐specific binding of the antibody to the SLC38A10 protein.

### Abundant immunostaining of SLC38A10 in mouse brain

**Figure 4:**
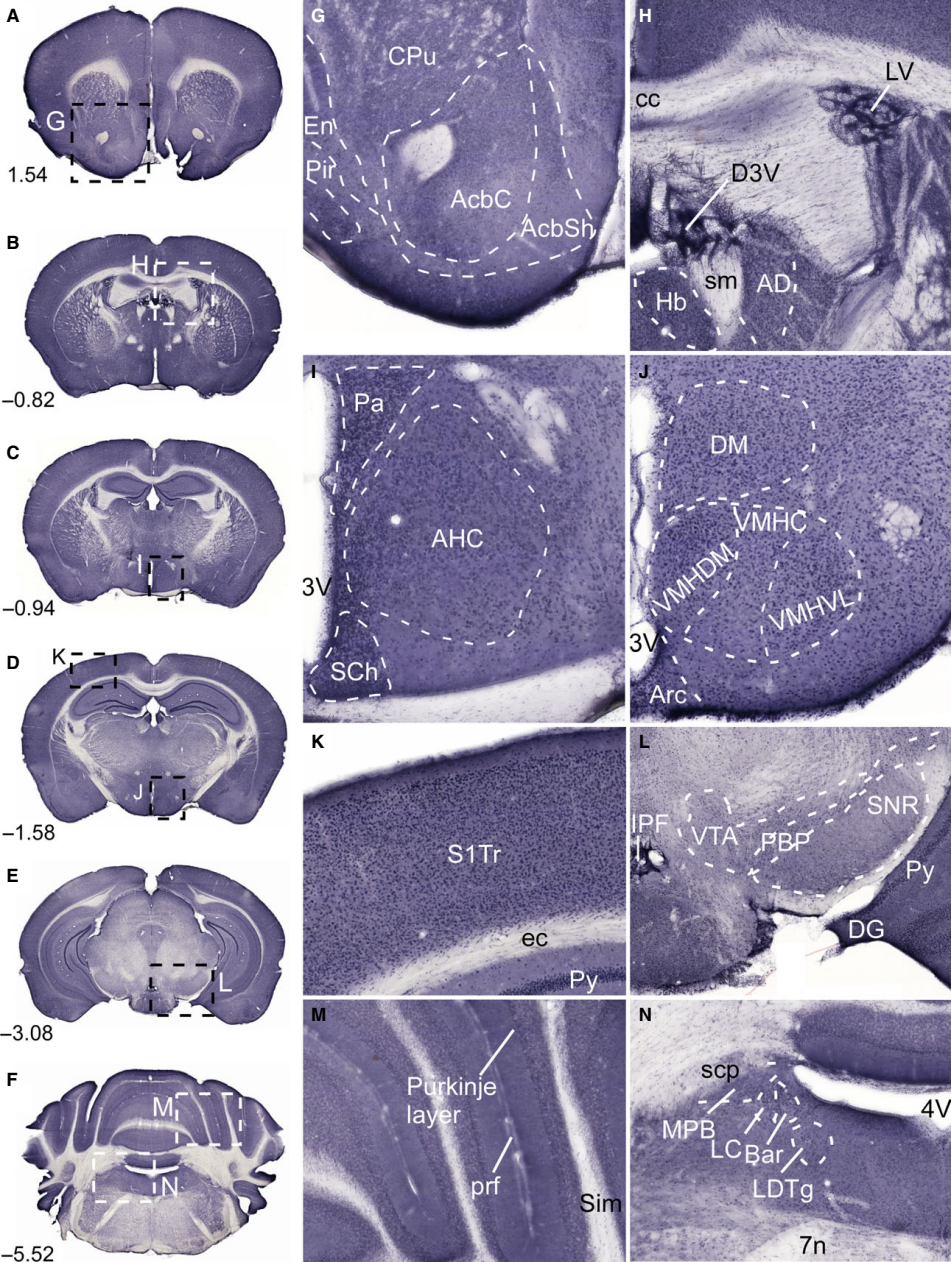
Immunostaining of SLC38A10 in the mouse brain. Nonfluorescent immunohistochemistry on free‐floating sections with overview pictures (A–F) and close up pictures (G–N) of SLC38A10 immunostaining in mouse brain. (G) Staining of SLC38A10 in nucleus accumbens (Acb), caudate putamen (CPu, striatum) and in piriform cortex (Pir) (Bregma 1.54). (H) SLC38A10 immunoreactivity in cells within D3V and LV (Bregma −0.82). (I) High immunostaining of SLC38A10 in paraventricular hypothalamic nucleus (Pa), suprachiasmatic nucleus (SCh) and in anterior hypothalamic area central part (AHC) close to the 3V (Bregma −0.94). (J) Localization of SLC38A10 in dorsomedial hypothalamic nucleus (DM), arcuate hypothalamic nucleus (Arc), and in ventromedial hypothalamic nucleus (VMH) (Bregma −1.58). (K) Scattered SLC38A10 staining in cells throughout cerebral cortex (Bregma −1.58). (L) Immunostaining of SLC38A10 in ventral tegmental area (VTA), dentate gyrus (DG), and in pyramidal cell layer of the hippocampus (Py) (Bregma −3.08). (M) SLC38A10 immunoreactivity in the Purkinje layer of cells in cerebellum (Bregma −5.52). (N) Immunostaining of SLC38A10 in locus coeruleus (LC) and Barrington's nucleus (Bar) in pons close to the 4V. Additional abbreviations: endopiriform claustrum (En), accumbens nucleus core (AcbC), accumbens nucleus shell (AcbSh), corpus callosum (cc), habenular nucleus (Hb), stria medullaris (sm), anterodorsal thalamic nucleus (AD), ventromedial hypothalamic nucleus central part (VMHC), ventromedial hypothalamic nucleus dorsomedial part (VMHDM), ventromedial hypothalamic nucleus ventrolateral part (VMHVL), primary somatosensory cortex trunk region (S1Tr), external capsule (ec), interpeduncular fossa (IPF), parabrachial pigmented nucleus of the VTA (PBP), substantia nigra reticular part (SNR), primary fissure (prf), simple lobule (Sim), superior cerebellar peduncle (scp), medial parabrachial nucleus (MPB), laterodorsal tegmental nucleus (LDTg), and facial nerve (7n). The described brain regions were depicted using, The Mouse Brain, by Franklin and Paxinos 2007.

A screen of the mouse brain was performed using nonfluorescent immunohistochemistry on free‐floating mouse brain sections, see Fig. 4. Extensive immunoreactivity of the transporter was detected in striatum, in ependyma, and cells close to the ventricles, cerebral cortex, hippocampus, hypothalamus, thalamus, pons, and cerebellum. Furthermore, SLC38A10 immunoreactivity was seen in accumbens nucleus (Acb) and piriform cortex (pir) (Fig. 4A and G). High immunostaining of SLC38A10 was located to cells within dorsal 3rd ventricle (D3V) and lateral ventricle (LV), possible choroid plexus (Fig. 4B and H). Hypothalamic staining of SLC38A10 was visualized in paraventricular hypothalamic area (Pa), suprachiasmatic nucleus (SCh) and in anterior hypothalamic area, central (AHC) (Fig. 4C and I). Additional hypothalamic staining was in dorsomedial hypothalamic nucleus (DM), arcuate nucleus (Arc), and in ventromedial hypothalamic nucleus (VMH) (Fig. 4D and J). SLC38A10 immunostaining was located to cells scattered throughout the cerebral cortex and in the pyramidal cell layer (Py) of the hippocampus (Fig. 4D and K). In the midbrain, SLC38A10 localized to ventral tegmental area (VTA) and dentate gyrus (DG) (Fig. 4E and L). In cerebellum SLC38A10 staining was detected in the Purkinje layer (Fig. 4F and M), and in pons, in locus coeruleus (LC) and Barringtons nucleus (Bar) close to the 4th ventricle (4V) (Fig. 4F and N).

### Neuronal, astrocytic, and epithelial localization of SLC38A10 in mouse CNS

**Figure 5:**
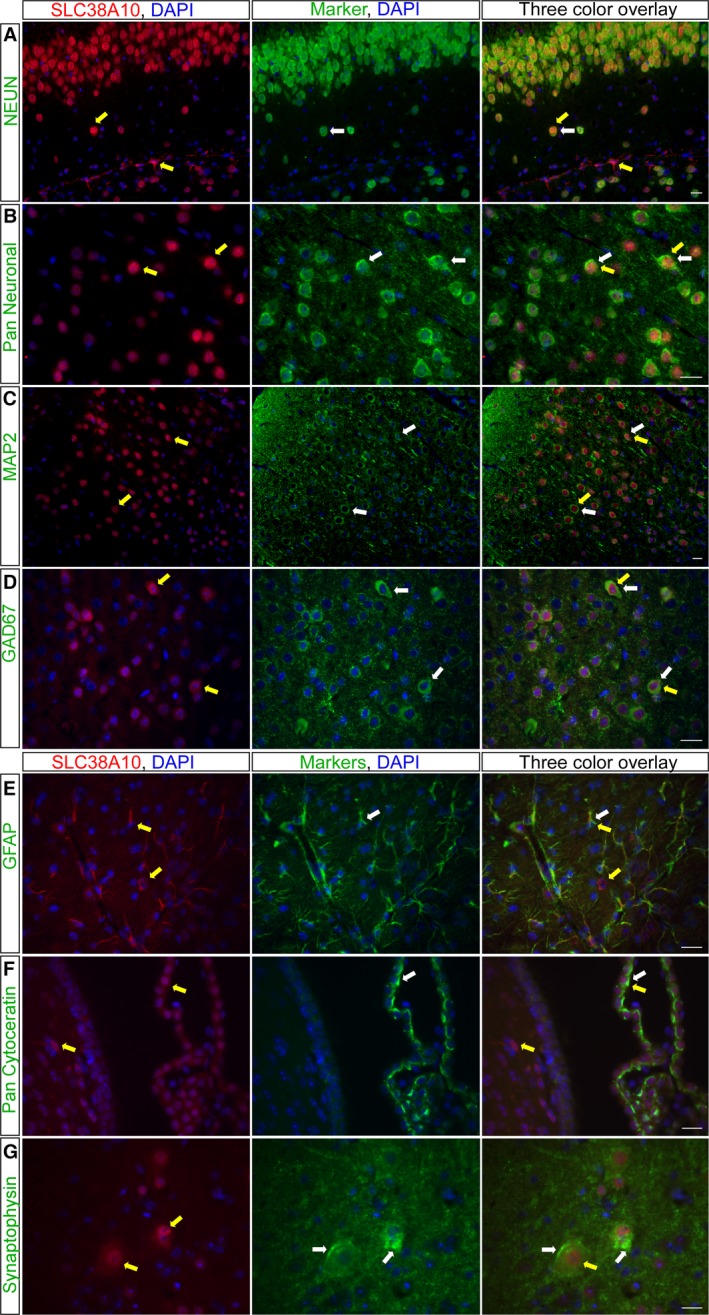
Immunostaining of SLC38A10 in neurons and astrocytes. Fluorescent immunohistochemistry on mouse brain and spinal cord sections with SLC38A10 immunoreactivity (red), protein markers (green), and nucleus marker DAPI (blue). (A) The neuronal marker NeuN co‐localized with SLC38A10 in hippocampus in brain (Bregma −2.06). (B) Overlapping staining between SLC38A10 and pan Neuronal in cortex in brain (Bregma 0.14). (C) The immunostaining of SLC38A10 and the neuronal marker MAP2 was overlapping in motor neurons in spinal cord (upper vertebrae L2 lumbar). (D) The SLC38A10 staining colocalized with cells labeled with the GABAergic marker Gad67 in cortex in brain (Bregma 0.26). (E) Highly overlapping immunostaining between SLC38A10 and the astrocyte marker GFAP in hypothalamus close to 3V in brain (Bregma 0.26). (F) Co‐localization of SLC38A10 and pan Cytokeratin in cells surrounding the LV in brain (Bregma −0.10). (G) No overlap between SLC38A10 and the synapse marker Synaptophysin in spinal cord (upper vertebrae L2 lumbar). Yellow arrows indicate cells expressing the SLC38A10 protein and white arrows indicate cells expressing the marker. Scale bars 20 μm.

Fluorescent immunohistochemistry was used to study colocalization of SLC38A10 immunoreactivity with antibody markers to pinpoint the type of cells expressing the transporter on paraffin‐embedded mouse brain and spinal cord sections (Fig. 5). A number of neuronal markers were used and overlapping staining was detected with the marker of neuron‐specific nuclear proteins of most neuronal cell types NeuN 44 in the CA fields of hippocampus in brain (Fig. 5A). The SLC38A10 immunostaining also colocalized with the neuronal cell marker pan Neuronal 45 in cerebral cortex in brain (Fig. 5B), and with the neuron‐specific cytoskeletal protein marker microtubule associated protein 2 (MAP2) 46 in large motor neurons and interneurons in the spinal cord (Fig. 5C). Moreover, the Gad67 marker was used for staining GABAergic neurons 47 and the immunoreactivity of SLC38A10 and Gad67 colocalized in inhibitory neurons in cerebral cortex in brain (Fig. 5D). Staining also colocalized in astrocytes, visualized with overlapping staining between the astrocyte marker glial fibrillary acidic protein (GFAP) 48 and SLC38A10 in hypothalamus close to the third ventricle (3V) in brain (Fig. 5E). In addition, SLC38A10 immunoreactivity was detected in cells with positive staining of the choroid plexus epithelial cell marker pan Cytokeratin 49 in cells surrounding the lateral ventricle (LV) in brain (Fig. 5F). The lack of colocalization between SLC38A10 and the vesicular marker Synaptophysin 50 suggests that SLC38A10 does not overlap with vesicles in nerve terminals in grey matter in the spinal cord (Fig. 5G). Taken together, SLC38A10 staining was located to neurons, astrocytes, and epithelial cells in mouse CNS.

### SLC38A10 transports l‐alanine, l‐glutamine, l‐glutamate, d‐aspartate, and MeAIB

**Figure 6:**
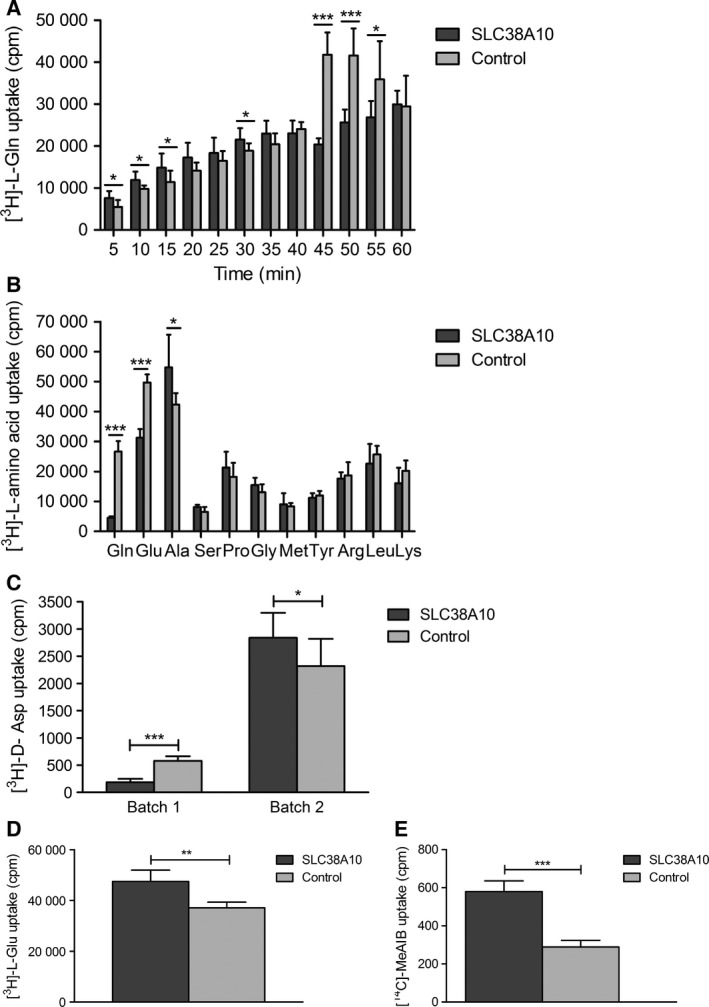
Transport data from radiolabeled uptake assays. The uptake of 0.2 μm radiolabeled substrate in SLC38A10 overexpressing oocytes compared with control oocytes was measured in counts per minute (cpm) using scintillation counting. Unpaired t‐test with 95% confidence interval was performed, (*P < 0.05, **P < 0.01, ***P < 0.001), (*n* = number of SLC38A10 overexpressing oocytes; number of control oocytes). (A) Uptake of l‐glutamine was measured every 5 min for 60 min, (measured uptake ± SD), [(5 min, *n* = 8;8, *P* = 0.0222), (10 min, *n* = 7;8, *P* = 0.0197), (15 min, *n* = 8;8, *P* = 0.0405), (20 min, *n* = 7;8, *P* = 0.0504), (25 min, *n* = 8;7, *P* = 0.2676), (30 min, *n* = 8;8, *P* = 0.0328), (35 min, *n* = 8;8, *P* = 0.1007), (40 min, *n* = 8;8, *P* = 0.4081), (45 min, *n* = 8;8, *P* < 0.0001), (50 min, *n* = 8;8, *P* < 0.0001), (55 min, *n* = 8;8, *P* = 0.0217), (60 min, *n* = 8;8, *P* = 0.8633)]. Uptake of l‐glutamine by SLC38A10 overexpressing oocytes was measured initially (5–30 min) while higher uptake was measured in controls later at 45–55 min. (B) Uptake assay screen of 11 amino acids with 50 min incubation time, (measured uptake ± SD), [Gln (l‐glutamine) *n* = 11;12, *P* < 0.0001], [Glu (l‐glutamatic acid), *n* = 6;6, *P* < 0.0001], [Ala (l‐alanine), *n* = 5;6, *P* = 0.0273], [Ser (l‐serine), *n* = 6;6, *P* = 0.0551], [Pro (l‐proline), *n* = 6;6, *P* = 0.3030], [Gly (glycine), *n* = 6;5, *P* = 0.1520], [Met (l‐methionine), *n* = 5;6, *P* = 0.7039], [Tyr (l‐tyrosine), *n* = 6;6, *P* = 0.4203], [Arg (l‐arginine), *n* = 6;5, *P* = 0.6040], [Leu (l‐leucine), *n* = 6;5, *P* = 0.3547] and [Lys (l‐lysine), *n* = 6;6, *P* = 0.1310]. (C) Competition uptake assay of d‐aspartate using two different batches of oocytes with 50 min incubation time, (measured uptake ±SD), Batch 1, *n* = 7;8, *P* < 0.0001, Batch 2, *n* = 11;12, *P* = 0.0170. (D) Additional uptake assay of l‐glutamate with 50 min incubation time, (measured uptake ±SD), (l‐Glu, *n* = 6;6, *P* = 0.0045). (E) Uptake of MeAIB with 50 min incubation time, (measured uptake±SD), *n* = 8;11, *P* = 0.0002.

To determine the substrate profile for SLC38A10 we used *X. laevis* oocytes to overexpress the transporter, and performed radiolabeled uptake assays (Fig. 6). This is a commonly used expression system for membrane‐bound transporters 21, 51. The uptake of l‐glutamine was measured every five min for 60 min and the oocytes were incubated in 0.2 μm [^3^H]‐l‐Gln and 1 μm nonlabeled l‐Gln in KRH buffer. The SLC38A10 overexpressing oocytes displayed significant uptake of l‐glutamine early, between 5–30 min while significantly higher uptake was measured in the controls at 45–55 min compared with SLC38A10 oocytes (Fig. 6A). Oocytes were incubated in KRH buffer with radiolabeled 0.2 μm [^3^H]‐l‐amino acid and 1 μm nonlabeled l‐amino acid for 50 min, and the uptake was measured. The uptake assay of 11 amino acids resulted in significantly higher uptake of l‐glutamine and l‐glutamate in controls compared with SLC38A10 oocytes while significant uptake of l‐alanine was measured in SLC38A10 oocytes compared with controls. There was no significant uptake of l‐serine, l‐proline, glycine, l‐methionine, l‐tyrosine, l‐arginine, l‐leucine, or l‐lysine (Fig. 6B). The uptake of d‐aspartate was measured at 50 min in competition with l‐aspartate (0.2 μm [^3^H]‐d‐Asp and 1 μm nonlabeled l‐Asp in KRH buffer). The uptake was measured in different batches of oocytes. Using the first batch (Batch 1) of oocytes significantly higher uptake of d‐aspartate was observed in controls, while the second batch (Batch 2) of oocytes resulted in significantly higher uptake of d‐aspartate in SLC38A10 oocytes compared with controls (Fig. 6C). An additional uptake assay using [^3^H]‐l‐Glu was run at 50 min which resulted in significant uptake of l‐glutamate in SLC38A10 oocytes (Fig. 6D). The synthetic amino acid analog MeAIB was significantly taken up by oocytes expression SLC38A10 (Fig. 6E).

### Efflux of l‐alanine, l‐glutamine, l‐glutamate, d‐aspartate, and l‐serine by SLC38A10

**Figure 7:**
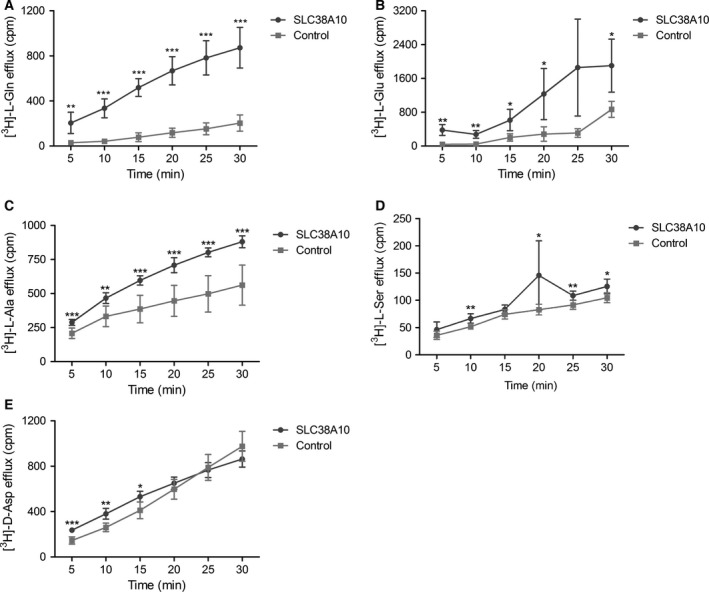
Transport data from radiolabeled efflux assays. The SLC38A10 oocytes and control oocytes were injected with 23 nL of radiolabeled amino acid and the efflux was measured in 100 μL of transport buffer every 5 min for 30 min in count per minute (cpm) using scintillation counting. Unpaired t‐test with 95% confidence interval was performed, (*P < 0.05, **P < 0.01, ***P < 0.001), (*n* = number of SLC38A10 overexpressing oocytes; number of control oocytes), (A) l‐glutamine efflux assay at pH 7.8, (measured efflux ±SD), *n* = 6;6, [(5 min, *P* = 0.0011), (10 min, *P* < 0.0001), (15 min, *P* < 0.0001), (20 min, *P* < 0.0001), (25 min *P* < 0.0001), (30 min, *P* < 0.0001)]. (B) l‐glutamate efflux assay at pH 7.8, (measured efflux ±SD), *n* = 4;4, [(5 min, *P* = 0.0049), (10 min, *P* = 0.004), (15 min, *P* = 0.0372), (20 min, *P* = 0.0398), (25 min *P* = 0.0584), (30 min, *P* = 0.0340)]. (C) l‐alanine efflux assay at pH 7.4, (measured efflux ±SD), *n* = 7;7, [(5 min, *P* = 0.0004), (10 min, *P* = 0.0014), (15 min, *P* = 0.0002), (20 min, *P* = 0.0001), (25 min *P* < 0.0001), (30 min, *P* = 0.0001)], (D) l‐serine efflux assay at pH 7.8, (measured efflux ±SD), [(5 min, *n* = 6;6, *P* = 0.1472), (10 min, *n* = 5;5, *P* = 0.0054), (15 min, *n* = 5;6, *P* = 0.1017), (20 min, *n* = 6;6, *P* = 0.0369), (25 min, *n* = 5;6, *P* = 0.00074), (30 min, *n* = 5;6, *P* = 0.0140)], (E) d‐aspartate efflux assay at pH 7.8. (measured efflux ±SD), *n* = 5 for both groups and times except *n* = 6 for controls at 5 and 10 min [(5 min, *P* = 0.0003), (10 min, *P* = 0.0011), (15 min, *P* = 0.0160), (20 min, *P* = 0.2821), (25 min *P* = 0.7074), (30 min, *P* = 0.1307)].

Since both higher and lower uptake was measured for several amino acids in SLC38A10 oocytes compared with controls, efflux assays was performed by injecting oocytes with 23 nL radiolabeled amino acid. The efflux was measured in 100 μL of ND96 transport buffer every 5 min for 30 min (Fig. 7). The efflux assay resulted in significant efflux of l‐glutamine (Fig. 7A), l‐glutamate (Fig. 7B), l‐alanine (Fig. 7C), l‐serine (Fig. 7D), and d‐aspartate (Fig. 7E). No significant efflux was measured for l‐proline, glycine, l‐methionine, l‐tyrosine, l‐arginine, l‐leucine, or l‐lysine (data not shown).

## Discussion

In this paper, we have used pairwise global sequence alignments to determine the sequence identity between the SLC38 family members. SLC38A10 was predicted to have 11 transmembrane helices with an intracellular N‐terminal and a 722‐amino acid long C‐terminal on the outside of the membrane. Furthermore, immunoreactivity of SLC38A10 was located to inhibitory neurons and other neurons, as well as astrocytes, and the staining was abundant in the mouse brain. We show that the previously uncharacterized protein SLC38A10 is an amino acid transporter, with preference for l‐glutamine, l‐alanine, l‐glutamate, d‐aspartate, and l‐serine.

The immunostaining of SLC38A10 was abundant in mouse brain. More specifically, in the hippocampus, staining of SLC38A10 was located in cells in the granular layer of dentate gyrus 52 and pyramidal cell layer of hippocampus, areas that support selective expression by excitatory neurons 53. Immunoreactivity was detected in the Purkinje cell layer in cerebellum, known to be GABAergic 54. In the brain, the previously studied SLC38 members are either expressed in neurons (SLC38A6 33, SLC38A7 24, SLC38A8 22, and SLC38A9 34) or astrocytes (SLC38A3 31 and SLC38A5 32), or in both neurons and glia (SLC38A1 27, 28 and SLC38A2 30). Here, we detected SLC38A10 immunoreactivity in neurons and astrocytes (Fig. 5), which resembles cell type localization for SLC38A1 and SLC38A2. We found this remarkable as SLC38A10 shared highest sequence identity with, SLC38A1 (23.1%) and SLC38A2 (24.7%), when looking at the TMH sequences. The SLC38A10 immunoreactivity was ubiquitous in astrocytes, while the neuronal staining was more cytoplasmic and more limited to the plasma membrane. In previous studies, intracellular immunoreactivity was detected for the plasma membrane‐bound transporters, SLC38A1 55 and SLC38A2 56, in the trans‐Golgi Network, where the proteins later are recruited from to the plasma membrane. It has also been demonstrated that SLC38A1 and SLC38A2 exerts their function in the plasma membrane of neurons 38. Adaptive regulation and the movement of the transporters within cells are features for both system A and system N transporters 57. Trafficking of transporters within the cell regulates transport activity and one stimulus, for example, the amino acid levels, can induce movement of transporters to the plasma membrane and increase transport activity, while another can decrease activity and withdraw the transporter from the plasma membrane 11, 58. Nevertheless, the specific subcellular localization of SLC38A10, or the recruitment of SLC38A10 to the plasma membrane must be further studied.

The results from the transport data suggest SLC38A10 to be a bidirectional transporter for l‐alanine, l‐glutamate, l‐glutamine, and d‐aspartate, while efflux was measured for l‐serine. When measuring the transport of glutamine using different incubation times (5–60 min), both significantly higher and lower uptake was measured in SLC38A10 oocytes, in a time‐dependent manner (Fig. 6A). These results indicated that SLC38A10 can mediate both uptake and efflux of glutamine. Bidirectional glutamine transport are reported for the system N transporters SLC38A3 17 and SLC38A5 59. In our uptake studies we used amino acid concentrations at 1.2 μm, which are high compared to the endogenous levels in oocytes, which are in the range of 0.07–3.6 nm 60. It is possible that the uptake and the putative efflux phases of glutamine are a result of altered gradients for ions and substrates over time, and that SLC38A10 has the capacity to control the balance between intracellular and extracellular concentrations, resulting in periods of uptake and efflux. Our transport experiments do not measure the SLC38A10 *K*_m_ values. However, the *K*_m_ values have been measured for other family members, for example the *K*_m_ value for glutamine is 0.3 mm for SLC38A1 27 and 1 mm for SLC38A5 59. Moreover, both lower and higher uptake of l‐glutamate was measured at 50 min for SLC38A10 oocytes compared with controls (Fig. 6B and D) using oocytes from different frogs. This phenomenon was observed several times, and the batch of oocytes affected the transport direction at a given time, although the pattern with increased and decreased uptake was consistent. This made it difficult to determine the substrate profile, by only rely on uptake assays, and therefore efflux assays were performed to substantiate the results. Efflux of l‐alanine, l‐glutamine, l‐glutamate, and d‐aspartate was measured, as well as for l‐serine. SLC38A10 can most likely facilitate uptake of l‐serine as well, even though our uptake studies failed to show this, probably because we did not time the uptake phase correctly. It is also possible that this transporter do transport l‐aspartate, since both uptake and efflux of d‐aspartate was observed in competition with l‐aspartate (Fig. 6C). The excitatory amino acid transporters (EAATs), from the SLC1 family, are nonselective toward l‐ and d‐aspartate, while selective for l‐glutamate 61. The system A hallmark substrate MeAIB was transported, however, SLC38A10 also shares the efflux features with system N and it can as well translocate charged amino acids, which complicated the system classification. Moreover, alanine and glutamine are the two most common substrates transported by the SLC38 family members 11, but this is the second member that translocates the charged amino acids glutamate and aspartate. SLC38A8 also transports MeAIB and was classified to system A, although it transported charged amino acids 22. Taken together, SLC38A10 transport the paradigm substrates for system A, glutamine, alanine, and serine, as well as MeAIB, and hence, we suggest it to be classified to system A.

In the GGGC, the SLC38 proteins are suggested to mediate uptake of glutamine in neurons and efflux of glutamine from astrocytes. SLC38A3 39 and SLC38A5 40 are suggested to facilitate the glutamine flux from astrocytes, which are qualities possibly also shared by SLC38A10. In addition, SLC1A5 is also suggested to mediate glutamine flux from astrocytes 62. SLC38A1 16, 27, 38, SLC38A2 38, SLC38A7 24, and SLC38A8 22 are suggested to enable glutamine uptake in the axon of neurons, close to the synaptic cleft. More specifically, SLC38A2 most likely facilitate uptake of glutamine in excitatory neurons 30, while this is mainly mediated by SLC38A1 in inhibitory neurons 55. Glutamine uptake can be facilitated by SLC38A7 24 and SLC38A8 22 in both inhibitory and excitatory neurons. In addition to immunoreactivity on axons, SLC38A7 24 and SLC38A8 22 are also intense on the soma, also suggesting a role in supplying neurons with glutamine for general biosynthesis. SLC38A10 has the capacity to facilitate glutamine uptake in neurons, and in addition mediate glutamine flux from astrocytes. It is also very remarkable that SLC38A10 transport the two most important excitatory amino acids in the brain, glutamate and aspartate. Therefore, SLC38A10 also have the capacity for glutamate uptake in neurons and astrocytes, following glutamate release from excitatory neurons, which are functions mediated by the EAATs 61. d‐serine function as a coagonist on the N‐methyl‐d‐aspartate (NMDA) receptor and the activation of the receptor requires binding of glutamate in combination with a coagonist. d‐serine is taken up by astrocytes and converted to l‐serine by the enzyme serine racemase (SR) 63. Although, l‐serine is also produced from glucose in glia cells 64. l‐serine is exported from astrocytes, a pathway suggested to be conducted by SLC1A4 65 and SLC1A5 66. Furthermore, SLC1A4 and SLC1A5 are also transporters for d‐serine in rat hippocampal astrocytes 67. Effluxes of l‐serine were measured for SLC38A10 and it is possible that SLC38A10 transport l‐serine from astrocytes, to serve neurons with l‐serine for d‐serine synthesis 64. Glycine is also a coagonist on the NMDA receptor and SLC38A5 is suggested to mediate the glycine flux from astrocytes 40. Nevertheless, the subcellular localization where SLC38A10 exert its function need to be further studied. SLC38A10 have the highest TMH sequence identity with SLC38A2, although the sequence identity was in equal proportions (21.9–24.7%) with SLC38A1, SLC38A3, SLC38A5, SLC38A7, and SLC38A8, and could thus have properties similar to them all, which supports our findings.

In conclusion, in the present study we show that SLC38A10 immunostaining is abundant in the mouse brain and the staining colocalized with markers for both neurons and astrocytes. We also show that the transporter is bidirectional and can mediate both uptake and efflux of l‐glutamine, l‐alanine, l‐glutamate, and d‐aspartate. In addition, efflux of l‐serine was measured. The results from the transport assays propose SLC38A10 to be classified into system A. Taken together, the cell type localization and the substrate profile for SLC38A10, suggests this transporter to have a potential role in pathways involved in both excitatory and inhibitory neurotransmission.

## Author contributions

SH wrote manuscript, planned and performed transport experiments, analysis and statistics of transport data, transmembrane predictions and sequence identity alignments, compiled figures. MH planned and analyzed IHC experiments and western blot, cloning, wrote the sections on western blot and IHC, compiled IHC and western blot figures. ME assisted with surgery of frogs, aided in analysis of transport data. RF drafted manuscript, planned experiments, conducted data analysis, conceived the study. All authors read and approved the final manuscript.
